# Variation in the Effect of Particulate Matter on Pulmonary Function in Schoolchildren in Western Japan and Its Relation with Interleukin-8

**DOI:** 10.3390/ijerph121114229

**Published:** 2015-11-09

**Authors:** Masanari Watanabe, Hisashi Noma, Jun Kurai, Hiroyuki Sano, Hiroya Kitano, Rumiko Saito, Yutaka Kimura, Setsuya Aiba, Mitsuo Oshimura, Eiji Shimizu

**Affiliations:** 1 Department of Respiratory Medicine and Rheumatology, Faculty of Medicine, Tottori University, 36-1 Nishi-cho, Yonago 683-8504, Japan; E-Mails: junkurajun@gmail.com (J.K.); eiji@med.tottori-u.ac.jp (E.S.); 2 Department of Data Science, Institute of Statistical Mathematics, 10-3 Midori-cho, Tachikawa, Tokyo 190-8562, Japan; E-Mail: noma@ism.ac.jp; 3 Department of Respiratory Medicine and Allergology, Faculty of Medicine, Kinki University, Ohnohigashi 377-2, Osakasayama 589-0014, Japan; E-Mail: hsano@med.kindai.ac.jp; 4 Board of Directors, Tottori University, 36-1 Nishi-cho, Yonago 683-8504, Japan; E-Mail: hkitano@med.tottori-u.ac.jp; 5 Department of Integrative Genomics, Tohoku Medical Megabank Organization, Tohoku University, 2-1 Seiryo-machi, Aoba-ku, Sendai 980-8573, Japan; E-Mail: rumiko-s@med.tohoku.ac.jp; 6 Department of Dermatology, Graduate School of Medicine, Tohoku University, 1-1 Seiryo-machi, Aoba-ku, Sendai 980-8574, Japan; E-Mails: yutakakimura@m.tohoku.ac.jp (Y.K.); saiba@med.tohoku.ac.jp (S.A.); 7 Chromosome Engineering Research Center, Tottori University, 36-1 Nishi-cho, Yonago 683-8504, Japan; E-Mail: oshimura@med.tottori-u.ac.jp

**Keywords:** interlukin-8, peak expiratory flow, PM_2.5_, schoolchildren, suspended particle matter

## Abstract

This study aimed to investigate the effects of particulate matter (PM) on pulmonary function in schoolchildren, as well as the relationships of these effects with interleukin-8. Morning peak expiratory flow (PEF) was measured daily in 399 children during April–May 2012, and in 384 of these children during March–May 2013. PEF’s association with the daily levels of suspended particulate matter (SPM) and PM < 2.5 μm (PM_2.5_) was estimated using a linear mixed model. Interleukin-8 promoter activity was assessed in THP-G8 cells stimulated by fallen PM collected at Tottori University Hospital during four periods (two in 2012 and two in 2013). An increase of 14.0 μg/m^3^ in SPM led to PEF changes of −2.16 L/min in 2012 and −0.81 L/min in 2013, respectively. An increment of 10.7 μg/m^3^ in PM_2.5_ was associated with PEF changes of −2.58 L/min in 2012 and −0.55 L/min in 2013, respectively. These associations were only significant in 2012. Interleukin-8 promoter activity was significantly higher in both periods of 2012 than in 2013. There was a significant association between pulmonary function in schoolchildren and daily levels of SPM and PM_2.5_, but this association may differ depending on the PM’s ability to elicit interleukin-8 production.

## 1. Introduction

Particulate matter (PM) is an important component of ambient air pollution. It is categorized based on particle size as PM_10_, PM_2.5_, or PM_0.5_, which represent median aerodynamic diameters of less than 10, 2.5, and 0.5 μm, respectively. Numerous epidemiological research studies have demonstrated that exposure to PM correlates with human health risks [1,2]. For example, the specialized cancer agency of the World Health Organization, the International Agency for Research on Cancer, has reported that exposure to PM increases risk of lung cancer [3]. Globally, ambient pollutants are now the third leading contributor to disability-adjusted life years associated with chronic respiratory disease [4].

Similarly, many epidemiological studies have demonstrated significant associations between PM and pulmonary function in children [5]. Short-term exposures to PM can reduce pulmonary function and increase respiratory symptoms, especially among children with respiratory diseases [6,7]. However, several studies have been unable to find an association between PM and pulmonary function in children with asthma [8,9]. A European multicenter study of asthmatic children failed to detect any consistent relationship between PM and short-term health effects, despite the wide ranges of climatic conditions and pollutant mixtures that were encountered across the sites [10].

PM is continuously affected by both stationary sources (e.g., power plants, industries, incinerators, and residential heating) and mobile sources (e.g., road traffic) [11,12,13]. It can also change size, morphology, phase state, and chemical composition via coagulation, condensation, and chemical reactions [14]. In one study, the inflammatory potential of ambient PM exhibited heterogeneity based on city and season [15]. Neutrophils migrate to the lung during acute inflammation induced by exposure to air pollutants [16]. One study found that exposure to air pollutants also increased both the concentration of interleukin (IL)-8 in bronchial lavage fluid and IL-8 mRNA expression in bronchial biopsy tissue from healthy participants [17]. Considering this previous evidence, the various adverse respiratory effects that are induced by exposure to PM may be related to the production of pro-inflammatory cytokines, and the presence or absence of cytokine production may depend on the particular composition of the PM.

In 2012, a study investigated the influence of air pollutants on pulmonary function (as assessed via peak expiratory flow [PEF]) in schoolchildren in western Japan. In 2013, we also conducted an extended survey to investigate the different influences of Asian dust storms on pulmonary function in children [18]. In the current study, we used the same cohort of schoolchildren who were surveyed in 2012 and 2013 to assess the effects of different PM on the pulmonary function of children who did and did not have asthma. For each year of data, we also used an IL-8 luciferase assay to investigate IL-8 promoter activity and its relationship with the detrimental effects of PM on pulmonary function.

## 2. Experimental Section

### 2.1. Participants

The study was performed in Matsue, which is the capital city of Shimane Prefecture and is located in Southwest Japan. The population of Matsue is about 200,000 and the area is 530.2 km². In March 2012, all fourth-grade students aged 8–9 years from four of the 35 elementary schools in Matsue were enrolled in the study. The four elementary schools were within 10 km of each other and all participants lived within 1 km of the schools. The children’s morning PEF values were monitored daily from March to May of 2012 and 2013. March 2012 was used as trial period to allow the children to familiarize themselves with the monitoring process.

**Figure 1:**
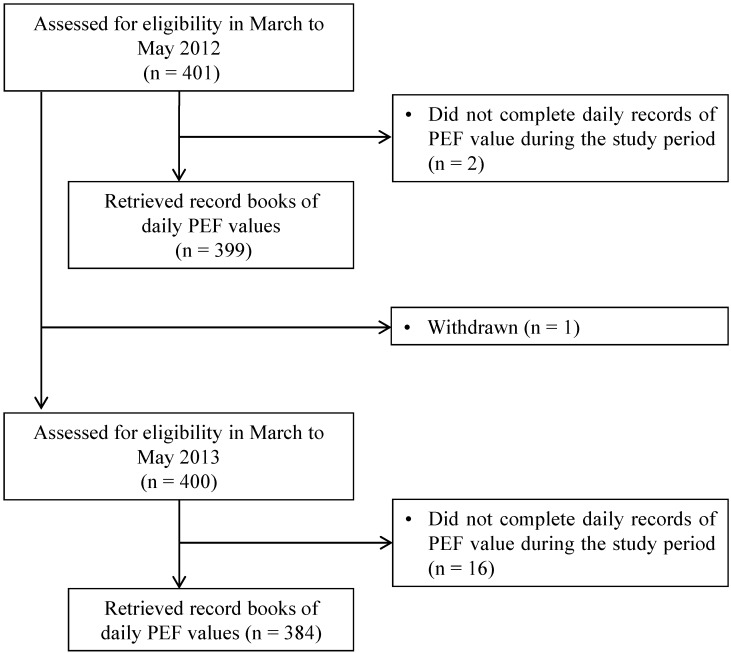
Flow chart showing the recruitment of the children in the study.

The recruitment process for 2012 and 2013 is shown in Figure 1. A total of 401 children were recruited into the study in March 2012. Two were subsequently excluded because of failures to keep the daily PEF records. Thus, records of daily PEF were analyzed for 399 children in 2012. In March 2013, we re-recruited the same 401 children, although one child was excluded because of Moyamoya disease. Sixteen children were subsequently excluded because of failures to keep the daily records of PEF value. Thus, records of daily PEF values were analyzed for 384 children in 2013.

In both March 2012 and March 2013, each participant was asked to record his or her age, sex, height, and weight, as well as the presence of asthma, allergic rhinitis, allergic conjunctivitis, atopic dermatitis, and food allergies. Participants were defined as having asthma if they met any of the following criteria in the past year: (1) diagnosis of asthma by a pediatrician; (2) wheezing; (3) use of asthma medication; or (4) regular visits to a hospital for asthma. Similarly, allergic rhinitis, allergic conjunctivitis, atopic dermatitis, and food allergy were judged to be present if the participants met any of the following criteria in the past year: (1) diagnosis by a pediatrician; (2) use of relevant medication for the disease; or (3) regular visits to a hospital for the disease.

The study was approved by the institutional ethics committee of Tottori University (Ethics Committee of Tottori University, Approval Number 1764). The Matsue City Board of Education approved and submitted the study proposal to the schools. The study was also approved by the Parent Teacher Association (PTA) of each participating elementary school. The children and their parents were informed by teachers and provided written consent.

### 2.2. Monitoring of PEF

Before the study, the children and their teachers were taught how to measure PEF values. All children then measured their morning PEF values daily using peak flow meters (Mini-Wright, Harlow, UK; American Thoracic Society scale) from March to May of 2012 and 2013, excepting weekends and public holidays. Each child was asked to record his or her greatest PEF value based on three attempts after arriving at school between 8 AM and 9 AM.

### 2.3. Measurement of Air Pollutant Levels

PM is classified into several categories according to size. PM_10_ is defined as any particle measuring less than l0 μm in diameter with a 50% cut-off, while PM_2.5_ as any particle measuring less than 2.5 μm in diameter with the same cut-off as PM_10_ [19]. In Japan, suspended particulate matter (SPM) is defined under the National Air Quality Standard as any particle with a diameter of less than 10 μm with a 100% cut-off [20]. The theoretical 50% cut-off diameter for SPM is assumed to be approximately 7 μm [20]. The particle diameter of SPM measurement in Japan is intermediate to the diameters that are used in the evaluation of PM_2.5_ and PM_10_. Although the daily fluctuations of SPM are similar to those of PM_2.5_ [19,20], the constituents of PM may differ across countries. The Japanese Ministry of the Environment monitors the levels of SPM instead of PM_10_. In Matsue City, the concentrations of SPM, PM_2.5_, sulfur dioxide (SO_2_), nitrogen dioxide (NO_2_), and ozone are also monitored by the Japanese Ministry of the Environment. These data were used to examine the relationships between changes in PEF values and air pollutant levels. Data on the daily temperature, humidity, and atmospheric pressure were obtained from the Japan Meteorology Agency in Matsue City. The observatories of the Japanese Ministry of the Environment and the Japan Meteorology Agency are both located within 10 km of the four elementary schools.

### 2.4. Preparation of the PM

Samples of fallen PM were collected four times at Tottori University Hospital, Yonago, which is located 30 km east of Matsue: (1) during April 7–April 20, 2012; (2) during April 26–May 10, 2012; (3) during April 8–22, 2013; and (4) during April 30–May 13, 2013. The collections were conducted with a large acrylic basin that had a collection area of 5,000 cm^2^ and a depth of 30 cm [21]. The collected samples of fallen PM were sterilized at 121 °C for 30 min in an autoclave (Tomy SX-300; Tomy Co., Tokyo, Japan) and stored in a freezer at −20 °C to prevent the growth of bacteria and fungi. For stimulation of the THP-G8 cells, the fallen PM was diluted to various concentrations with distilled deionized water.

### 2.5. IL-8 Promoter-Luciferase Gene Reporter Assay

THP-G8 cells are a THP-1-derived reporter cell line that expresses stable luciferase orange (SLO) and stable luciferase red (SLR) genes under the control of the IL-8 and glyceraldehyde 3-phosphate dehydrogenase (GAPDH) promoters, respectively [22]. The THP-G8 cell line was cultured as described previously [22]. Luciferase activity (LA) was determined using a microplate luminometer with a Phelios multicolor detection system (Atto Corp., Tokyo, Japan) using Tripluc luciferase assay reagent (Toyobo Co., Osaka, Japan). IL-8 promoter activity was assessed from normalized SLO luciferase activity (nSLO-LA), which was calculated as SLO-LA divided by SLR-LA, and the fold induction of nSLO-LA was calculated as the nSLO-LA level of treated cells divided by that of untreated cells [22]. The induction of IL-8 promoter activity was measured by comparing THP-G8 cells (5 × 104 cells/100 μL/well) in 96-well black plates (Greiner Bio-One GmbH, Frickenhausen, Germany) stimulated for 5 h with (a) solvent only (negative control); (b) 100 ng/mL lipopolysaccharide (LPS; positive control); or (c) 1 mg/mL PM collected in 2012 and 2013.

### 2.6. Statistical Analyses

To estimate the effect of exposures to SPM and PM_2.5_ on the children’s daily PEF values, we used linear mixed models that accounted for correlations among repeated measurements within the same participant [23,24]. The linear mixed models included a random intercept for the participants in the analysis. Additionally, the participants’ individual characteristics (sex, height, weight, asthma, allergic rhinitis, allergic conjunctivitis, atopic dermatitis, and food allergy), gaseous air pollutants (SO_2_, NO_2_, and ozone), and meteorological variables (daily temperature, humidity, and atmospheric pressure) were modeled as potential confounding factors. In our results, estimates are presented as the absolute difference in PEF values per interquartile range (IQR) change in exposure, and are accompanied by 95% confidence intervals (CIs). Linear mixed model analyses were performed using R version 3.0.3 (R Foundation for Statistical Computing, Vienna, Austria). Differences in the nSLO-LA of THP-G8 cells were evaluated using analysis of variance (ANOVA) in SPSS statistical software (Japanese ver. 21.0 for Windows; IBM Japan, Tokyo, Japan). All quoted *p* values are two-sided and the significance levels of all tests were set to 0.05.

## 3. Results

### 3.1. Characteristics of the Children

The characteristics of the children in the 2012 and 2013 studies are shown in Table 1.

**Table 1:** Characteristics of the schoolchildren included in the study.

	2012	2013
*Number*	399	384
Boy/Girl	205/194	194/190
*Height (cm)*	132.3 ± 5.9	137.7 ± 7.0
Boy	132.2 ± 5.5	136.9 ± 6.3
Girl	132.4 ± 6.4	138.5 ± 7.7
*Weight (kg)*	29.5 ± 5.8	32.4 ± 6.6
Boy	29.6 ± 6.2	32.3 ± 6.8
Girl	29.3 ± 5.4	32.6 ± 6.4
*Allergic disease (number)*		
Asthma	38	45
Allergic rhinitis	78	74
Allergic conjunctivitis	8	15
Atopic dermatitis	44	36
Food allergy	19	20

Data are shown as the mean ± standard deviation.

### 3.2. PEF

**Figure 2:**
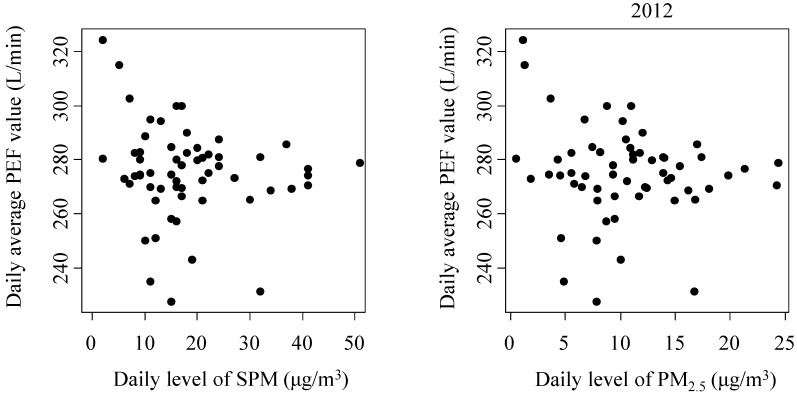

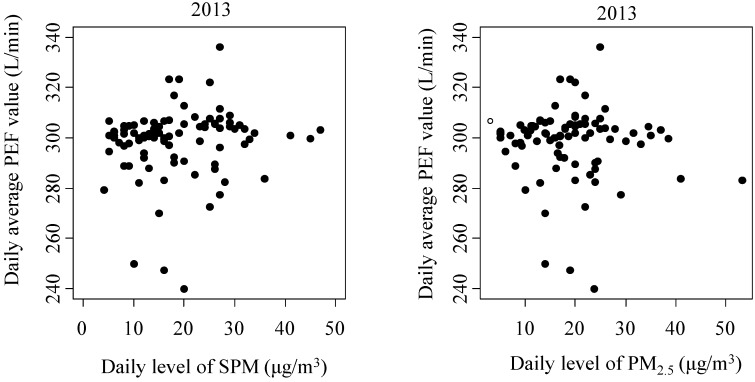
The associations of daily average peak expiratory flow (PEF) values and daily levels of suspended particulate matter (SPM) and particulate matter smaller than 2.5 μm in diameter (PM_2.5_) in the 2012 and 2013 surveys.

Results of the estimated changes in PEF values for interquartile range (IQR) increases in exposure to SPM and PM_2.5_ are presented in Table 2. In the 2012 survey, SPM was significantly associated with PEF in both children who did and did not have asthma, with an increase of 14.0 μg/m^3^ in SPM reducing the PEF value by 2.16 L/min (−2.06 L/min in children without asthma and −3.11 L/min in children with asthma). Similarly, PM_2.5_ was negatively associated with the PEF value, with a 10.7 μg/m^3^ increase in PM_2.5_ decreasing the PEF value by 2.58 L/min in the total study cohort (−2.46 L/min in children without asthma and -3.69 L/min in children with asthma). In the 2013 survey, increases of 14.0 μg/m^3^ in SPM and 10.7 μg/m^3^ in PM_2.5_ led to changes in the PEF value of −3.41 L/min and −2.42 L/min in children with asthma, respectively. However, in 2013, there was no significant association between the PEF value and either SPM or PM_2.5_ for the total study cohort or for children without asthma. Figure 2 shows the relationship between the daily average PEF value and the daily levels of SPM and PM_2.5_.

**Table 2:** Associations of PEF values with interquartile increases in SPM and PM_2.5_ in linear mixed-effects models for the 2012 and 2013 surveys.

Year	Exposure Metric	IQR	All Children		
			Change in PEF Value (L/min)	95%CI	*p* Value
2012	SPM	14.0 μg/m^3^	−2.16	−2.88, −1.43	<0.0001
	PM_2.5_	10.7 μg/m^3^	−2.58	−3.59, −1.57	<0.0001
2013	SPM	14.0 μg/m^3^	−0.81	−1.68, 0.06	0.068
	PM_2.5_	10.7 μg/m^3^	−0.55	−1.30, 0.19	0.146
**Year**	**Exposure Metric**	**IQR**	**Children without Asthma**		
			**Change in PEF Value (L/min)**	**95%CI**	***p* Value**
2012	SPM	14.0 μg/m^3^	−2.06	−2.81, −1.30	<0.0001
	PM_2.5_	10.7 μg/m^3^	−2.46	−3.51, −1.41	<0.0001
2013	SPM	14.0 μg/m^3^	−0.44	−1.37, 0.47	0.337
	PM_2.5_	10.7 μg/m^3^	−0.29	−1.07, 0.49	0.464
**Year**	**Exposure Metric**	**IQR**	**Children with Asthma**		
			**Change in PEF Value (L/min)**	**95%CI**	***p* Value**
2012	SPM	14.0 μg/m^3^	−3.11	−5.70, −0.54	0.018
	PM_2.5_	10.7 μg/m^3^	−3.69	−7.28, −0.10	0.044
2013	SPM	14.0 μg/m^3^	−3.41	−6.11, −0.70	0.014
	PM_2.5_	10.7 μg/m^3^	−2.42	−4.70, −0.13	0.039

IQR: interquartile range; CI: confidence interval; PEF: peak expiratory flow; SPM: suspended particulate matter; PM_2.5_: particulate matter smaller than 2.5 μm in diameter.

**Figure 3:**
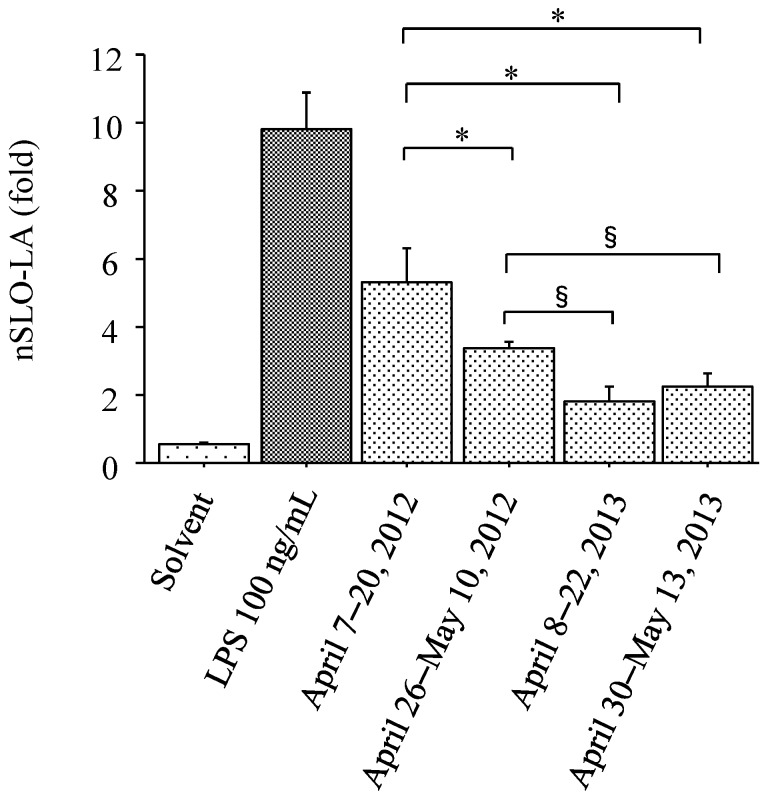
Interleukin-8 transcriptional activity measured using an interleukin -8 luciferase assay in a stable THP-1-derived interleukin-8 reporter cell line. Interleukin-8 transcriptional activity is based on normalized stable luciferase orange luciferase activity (nSLO-LA), which was calculated as stable luciferase orange luciferase activity divided by stable luciferase red luciferase activity. Cells were treated with solvent only (n = 6, negative control), lipopolysaccharide (LPS) (n = 6, 100 ng/mL, positive control), or particulate matter collected on: April 7–20, 2012 (n = 6, 1 mg/mL); April 26–May 10, 2012 (n = 6, 1 mg/mL); April 8–22, 2013 (n = 6, 1 mg/mL); and April 30–May 13, 2013 (n = 6, 1 mg/mL). * *p* < 0.0001 for the comparison with particulate matter collected during April 7–20, 2012. § *p* < 0.005 for the comparison with PM collected during April 26–May 10, 2012.

### 3.3. IL-8 Promoter Activity in THP-G8 Cells

The nSLO-LA values (IL-8 promoter activity) of THP-G8 cells (Figure 3) changed by 0.53 ± 0.05 fold (solvent, n = 6), 9.79 ± 1.10 fold (LPS, n = 6, 100 ng/mL), 5.32 ± 1.01 fold (April 7–20, 2012, n = 6, 1 mg/mL), 3.35 ± 0.22 fold (April 26–May 10, 2012, n = 6, 1 mg/mL), 1.82 ± 0.40 fold (April 8–22, 2013, n = 6, 1 mg/mL), and 2.23 ± 0.38 fold (April 30–May 13, 2013, n = 6, 1 mg/mL). Both of the collected PM samples from 2012 differed from the two 2013 samples in terms of the nSLO-LA values in THP-G8 cells (Figure 3). After stimulation by the fallen PM, the viability of the THP-G8 cells exceeded 95% in all four samples, as assessed using a trypan blue-exclusion test.

### 3.4. Supplementary Results

In addition to the analyses described above, we evaluated the sex-specific relationships of PEF with SPM and PM_2.5_; however, our results showed no interaction effect between the changes in PEF values and the participants’ sexes (Supplementary File, Table S1). In a second supplementary analysis, we evaluated IL-8 promoter activity in THP-G8 cells stimulated with 0.2 mg/mL, 0.4 mg/mL, and 1 mg/mL of collected PM. As expected, we found that nSLO-LA increased in a concentration-dependent manner (Supplementary File, Figure S1).

## 4. Discussion

Although many studies have clearly shown that exposure to PM is significantly associated with pulmonary function [5,6,7], several other studies have failed to find a consistent relationship [8,9,10]. In Japan, only been a limited number of studies have investigated the association between PM and pulmonary function. We conducted an extended survey to reassess the relationship between PM and pulmonary function in schoolchildren in Japan. In both 2012 and 2013, there were significant associations between pulmonary function and both SPM and PM_2.5_ in children with asthma. However, we were unable to find the same relationship between pulmonary function and SPM or PM_2.5_ in children without asthma, even though these children belonged to the schools and grades as the children with asthma. The effects of PM on the production of pro-inflammatory cytokines differed according to the period of collection. The fallen PM that had been collected in 2012 induced significantly greater IL-8 promoter activity than did the PM that had been collected in 2013. These results suggest that the effect of PM on pulmonary function in children may be mediated by the extent of airway inflammation.

Previous studies of PM and pulmonary function have shown heterogeneous results [6,7,8,9,10]. Although some of these differences may be attributable to differences in study design, methods, or data analysis, such factors are thought to account for only a small portion of the variation between study results [7,10,25,26]. The present study was conducted using methods that are similar to those employed in other studies, and the differences in study design, methods, and data analysis cannot account for the difference between the year 2012 and year 2013 effects of PM in children without asthma. Therefore, other, more substantial factors are likely to explain these discordant results.

A large proportion of fine and ultrafine PM has anthropogenic origins (e.g., emissions from combustion and motor vehicles) [27]. Ambient coarse PM and sand dust can also include components of geological origin [27,28]. The acute effects that are triggered by short-term exposure to particles can exacerbate inflammatory responses, and inflammation is associated with the long-term development of lung disease [29,30,31]. Dusts of geological origin are known to induce the production of pro-inflammatory cytokines [32]. Kumar *et* *al*. showed that ambient PM was more important than traffic-derived PM as a cause of injury to airway epithelial cells leading to the production of pro-inflammatory cytokines [33]. Therefore, in this study, we assessed and compared the potential production of IL-8 by fallen PM that had been collected in 2012 and 2013. Both PM samples collected during 2012 increased the levels of IL-8 significantly more than did the two samples from 2013. This could explain why the pulmonary function of children without asthma was not associated with SPM or PM_2.5_ in 2013. These results suggest that the association between PM and reduced pulmonary function depends on the ability of the PM to cause the production of pro-inflammatory cytokines.

The heavy dust emissions that originate from the deserts in East Asia have produced the second largest dust emissions worldwide. These dust storms have been referred to as Asian dust storms (ADS) [34]. ADS include high counts of aerosolized air pollutants in addition to mineral dust particles [35]. These sand emissions are known to induce the production of pro-inflammatory cytokines [32]. Therefore, in order to avoid the effects of ADS airborne particles on the secretion of IL-8, we collected PM during study periods that did not include days of ADS exposure. Additionally, the collection periods differed between 2012 and 2013.

Asthma is usually characterized by chronic airway inflammation [36]. It is defined by a history of respiratory symptoms such as wheezing, shortness of breath, chest tightness, and cough that vary over time and in intensity, along with variable expiratory airway limitations. Additionally, asthma is usually associated with airway hyperresponsiveness. Airway hyperresponsiveness itself usually persists, even when symptoms are absent and lung function is normal. Both airway limitation and respiratory symptoms are often triggered by various factors. Irritant exposure is one common trigger for impaired pulmonary function and respiratory symptoms in patients with asthma. Therefore, in this study, we performed subgroup analyses for the associations of pulmonary function with SPM and PM_2.5_, stratifying the children according to asthma status. The children with asthma exhibited associations between pulmonary function and SPM and PM_2.5_ in both 2012 and 2013.

There is a relationship between atopic disposition and airway hyperresponsiveness in children [37]. The influences of pollutants on one’s airway may differ depending on whether allergic diseases are present. Therefore, we also adjusted for allergic diseases in our analysis of the associations between air pollutants and PEF. In addition, the participants’ sex may influence the risk of wheezing and the prevalence of asthma throughout childhood [38]. We evaluated the sex-specific relationships of asthma with PEF; however, our results showed no interaction effect between the changes in PEF values and the participants’ sexes (Supplementary File).

To investigate the potential of PM to elicit production of IL-8, this study used samples of fallen PM. Experimental studies have repeatedly found that coarse PM is the most injurious to cells, even when other size fractions are delivered at the same mass or concentration [15,39]. However, several studies have reported contrasting findings—specifically that the associations between chemical compositions and particle toxicity tend to be stronger for fine and ultrafine PM [40,41]. Collectively, these findings show that the effects of PM on the production of pro-inflammatory cytokines can vary considerably, and that the composition of PM affects the observed differences. Future studies should measure the potentials of SPM and PM_2.5_ to elicit pro-inflammatory cytokines.

In a previous study, we investigated IL-8 transcriptional activity using an IL-8 promoter luciferase assay in THP-G8 cells (the so-called IL-8 Luc assay). We found that IL-8 transcriptional activity correlated significantly with the secretion of IL-8 [18]. At present, it is extremely difficult to collect sufficient quantities of PM and to separate it according to defined aerodynamic diameters. Smaller PM is especially difficult to collect. We used the IL-8 Luc assay in this study because it has high sensitivity for the evaluation of IL-8 levels using a small amount of material.

There are several limitations to the present study. First, we defined asthma based on the parents’ reports and were unable to diagnose asthma on the basis of airway hyperresponsiveness to methacholine and reversible airflow limitation. Therefore, the number of children with asthma differed between 2012 and 2013. Several children may have been incorrectly diagnosed with asthma. An over-diagnosis bias would have resulted in underestimations of the associations between PEF values and SPM and PM_2.5_. Second, missing PEF values due to school absences were excluded from the data analysis. However, this intermittent missing data was statistically independent and did not cause any serious bias in the results. Third, the study periods were limited. In particular, we were unable to assess any seasonal variation in the association of pulmonary function with SPM and PM_2.5_. Fourth, this study was unable to analyze the components and size distribution of collected PM. Therefore, it remains unclear which specific substance have substantial effects on decreases in pulmonary function and induction of IL-8. Fifth, we were unable to measure the individual amounts and durations of exposure to SPM and PM_2.5_. To the extent that we were able to confirm the relevant information from teachers, the educational curricula of 2012 and 2013 did no lead to differences in outdoor activities. The differences in the individual amounts and durations of exposure to SPM and PM_2.5_ may not been substantial, at least on school days. Finally, in the course of our investigation of the effects of PM on pulmonary function and induction of IL-8, we were unable to estimate the IL-8 concentrations in sputum and blood. Therefore, further study is needed to investigate these effects.

## 5. Conclusions

We found a significant association between pulmonary function in schoolchildren and daily levels of SPM and PM_2.5_. However, the effects of SPM and PM_2.5_ on pulmonary function may differ depending on the production of pro-inflammatory cytokines induced by PM.

## Supplementary Files

Supplementary File 1

## Abbreviations

ADS	Asian dust storm
ANOVA	analysis of variance
CI	confidence intervals
IL	interleukin
GAPDH	glyceraldehyde 3-phosphate dehydrogenase
IQR	interquartile range
LPS	lipopolysaccharide
nSLO-LA	normalized stable luciferase orange luciferase activity
PEF	peak expiratory flow
PM	particulate matter
PM_10_	particulate matter smaller than 10 μm
PM_2.5_	particulate matter smaller than 2.5 μm
PM_0.5_	particulate matter smaller than 0.5 μm
PTA	Parent Teacher Association
SD	standard deviation
SLO	stable luciferase orange
SLR	stable luciferase red
SPM	suspended particle matter
