# Supplementary material for: Variation in the Effect of Particulate Matter on Pulmonary Function in Schoolchildren in Western Japan and Its Relation with Interleukin-8
Source: Int J Environ Res Public Health. 2015 Nov 9;12(11):14229–43. doi: 10.3390/ijerph121114229 (PMC4661643; doi:10.3390/ijerph121114229)
Supplement: Supplementary File 1 [file ijerph-12-14229-s001.pdf]

Supplementary Information

Variation in the Effect of Particulate Matter on Pulmonary Function in Schoolchildren in Western Japan and Its Relation with Interleukin-8

1. Associations between Peak Expiratory Flow and Particulate Matter, as Stratified by Sex

In addition to the analyses presented in the main text, we estimated the sex-specific changes in the peak expiratory flow (PEF) value per increases of 1 interquartile range (IQR) in exposure to suspended particulate matter (SPM) and particulate matter smaller than 2.5 μm in diameter ($PM_{2.5}$). The results are presented separately for boys and girls in Table S1. In the 2012 survey, SPM was significantly associated with the PEF in both boys and girls: an increase of 14.0 μg/m^3 in SPM reduced PEF by 2.94 L/min in boys and 2.62 L/min in girls. Similarly, $PM_{2.5}$ was negatively associated with the PEF value: a 10.7 μg/m^3 increase in $PM_{2.5}$ decreased the PEF value by 4.06 L/min in boys and 3.55 L/min in girls. However, there was no significant difference between the changes in PEF values for boys and girls. In 2013, there was no significant association between the PEF value and either SPM or $PM_{2.5}$ in either boys or girls.

Table S1. Sex-specific associations of PEF values with interquartile increases of SPM and $PM_{2.5}$ in linear mixed-effects models.

Year	Exposure Metric	IQR	Boy		
			Change in PEF Value (L/min)	95%CI	*p* Value
2012	SPM	14.0 μg/m^3	−2.94	−4.22, −1.67	< 0.0001
	$PM_{2.5}$	10.7 μg/m^3	−4.06	−6.09, −2.03	< 0.0001
2013	SPM	14.0 μg/m^3	−0.90	1.94, 0.14	0.0911
	$PM_{2.5}$	10.7 μg/m^3	−0.50	−1.39, 0.40	0.2727
**Year**	**Exposure Metric**	**IQR**	**Girl**		
			**Change in PEF Value (L/min)**	**95%CI**	***p* Value**
2012	SPM	14.0 μg/m^3	−2.62	−4.03, −1.21	< 0.0001
	$PM_{2.5}$	10.7 μg/m^3	−3.55	−5.77, −1.34	0.0016
2013	SPM	14.0 μg/m^3	−0.71	−2.11, 0.68	0.3151
	$PM_{2.5}$	10.7 μg/m^3	−0.60	−1.79, 0.59	0.3231

IQR: interquartile range; CI: confidence interval; PEF: peak expiratory flow; SPM; suspended particulate matter; $PM_{2.5}$: particulate matter smaller than 2.5 μm in diameter.

2. Interleukin-8 Promoter Activity in THP-G8 Cells

When THP-G8 cells were stimulated with 0.2 mg/mL, 0.4 mg/mL, and 1 mg/mL of each collected particulate matter (PM) (n = 6), normalized stable luciferase orange luciferase activity values increased in a concentration-dependent manner (Figure S1).

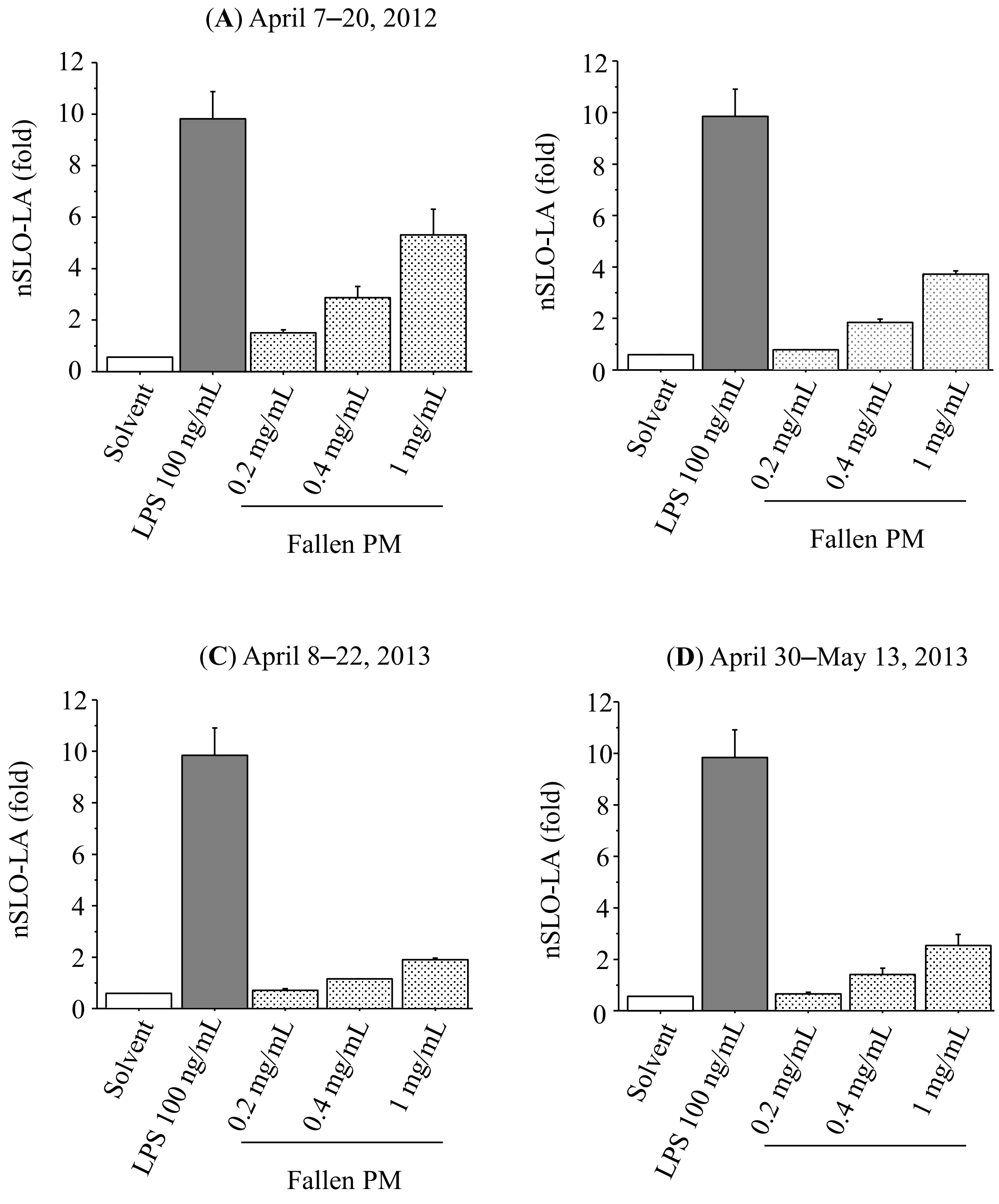

Figure S1. Interleukin-8 transcriptional activity in THP-G8 cells stimulated with solvent only (n = 6, negative control), LPS (n = 6, 100 ng/mL, positive control), or collected PM at 0.2 mg/mL, 0.4 mg/mL and 1 mg/mL (n = 6). Fallen PM collected on: (**A**) April 7–20, 2012; (**B**) April 26–May 10, 2012; (**C**) April 8–22, 2013; and (**D**) April 30–May 13, 2013. nSLO-LA indicates normalized stable luciferase orange luciferase activity.

© 2015 by the authors; licensee MDPI, Basel, Switzerland. This article is an open access article distributed under the terms and conditions of the Creative Commons Attribution license (http://creativecommons.org/licenses/by/4.0/).
